# Isolation and Characterization of Microsatellite Markers for *Passiflora contracta*

**DOI:** 10.3390/ijms130911343

**Published:** 2012-09-12

**Authors:** Ana Luíza R. Cazé, Raquel A. Kriedt, Luciano B. Beheregaray, Sandro L. Bonatto, Loreta B. Freitas

**Affiliations:** 1 Laboratory of Molecular Evolution, Department of Genetics, Federal University of Rio Grande do Sul, PO Box 15053, 91501-970 Porto Alegre, RS, Brazil; E-Mails: analuizacaze@gmail.com (A.L.R.C.); raquelkriedt@yahoo.com (R.A.K.); 2 Molecular Ecology Laboratory, School of Biological Sciences Flinders University, GPO Box 2100, Adelaide 5001, Australia; E-Mail: luciano.beheregaray@flinders.edu.au; 3 Laboratory of Genomic and Molecular Biology, Pontifical Catholic University of Rio Grande do Sul, Ipiranga 6681, 90610-001 Porto Alegre, RS, Brazil; E-Mail: slbonatto@pucrs.br

**Keywords:** nuclear microsatellites, *Passiflora contracta*, conservation genetics

## Abstract

*Passiflora contracta* Vitta (Passifloraceae) is an endemic species of the Atlantic Rainforest, one of the most species-rich ecoregions in the world, although extremely endangered. We have developed an enriched microsatellite library in order to fine-scale studies of the genetic structure of *P. contracta*. Twelve pairs of microsatellite primers were designed, and seven loci were successfully amplified and characterized by genotyping two wild populations of *P. contracta*. All seven loci were polymorphic, with an average number of alleles found being 4.8 and 5 per population. The cross-species transferability was tested using sister species *Passiflora ovalis* Vell. Ex Roemer. The development of these markers will contribute to the studies of population genetics in *P. contracta* as well as future studies concerning diversity patterns in the Atlantic Rainforest, and may also help to establish strategies for the conservation of this species.

## 1. Introduction

*Passiflora contracta* Vitta (Passifloraceae) is an endemic species of the Atlantic Rainforest, one of the most species-rich ecoregions in the world. Originally covering 15% of Brazil, this endangered ecosystem has been reduced to less than 8% of its coverage. Despite the anthropic interference, the Atlantic Rainforest still harbors impressively high levels of endemism and diversity [1], characteristic of areas of long-term climate stability, also known as refugia [2]. Although refugia have been proposed in the Atlantic Rainforest [3], these have not been tested by studies of the intraspecific plant genetic diversity due to the lack of information on the genetic diversity of the Atlantic Forest’s vegetation. *P. contracta* is an excellent species for such a study. This woody vine is distributed along the coast of Brazil, ranging from the Pernambuco to Espirito Santo states (~07–21°S latitude) [4]. The species is characterized by its chiropterophily syndrome, which is not a common feature in the Passifloraceae family [5].

The identification of high-resolution genetic markers within *P. contracta* is an important step to develop fine-scale investigations of endemism-rich ecosystems and is also an interesting tool for testing the refugia hypothesis. Therefore, the aim of this study was to develop and characterize microsatellite markers in *P. contracta* and to test their transferability to its sister species, *Passiflora ovalis* Vell. Ex Roemer (Passifloraceae).

## 2. Results and Discussion

A total of seven of the 12 primer pairs were successfully amplified; all of them were polymorphic and presented alleles in the expected size range for the two wild populations: Linhares-ES (19°24′S, 40°28′W) and Maraú-BA (14°07′50″S, 38°59′55″W) (Table 1). The characteristics of the microsatellite loci and variability measures across the two wild populations are described in Table 2. The number of alleles per population ranged from two to nine (Linhares) and from three to eight (Maraú). The average alleles found for each population was 5 and 4.8, respectively. The observed and expected heterozygosity ranged from 0.31 to 0.84 (Linhares), and 0.38 to 0.67 (Maraú). Two loci, PC6F7 (Linhares) and PC7H11 (Maraú), showed significant deviations from the Hardy-Weinberg equilibrium (*p* < 0.007). These findings may be a consequence of the high inbreeding in both populations, which could result from habitat fragmentation since species populations are small and, in general, restricted to preserved areas. Alternatively, the HWE deviation could be due to null alleles, which were indicated by MICRO-CHECKER 2.2.3 for the deviating loci (PC6F7) of the Linhares population, and also for the deviating loci (PC7H11) of the Maraú population. Null alleles were not detected for the remaining loci. One pair of loci (PC5E11 and PC6G11) showed significant linkage disequilibrium for the Linhares population after Bonferroni correction (*p* < 0.002). However, with no additional information, the physical linkage of the loci cannot be distinguished from disequilibrium due to population processes as nonrandom mating [6].

The transferability of the markers to the sister species *P. ovalis* was tested for all 12 primer pairs, and showed a low efficiency, with just two loci (PC6G11 and PC7H11) being amplified. This result reinforces the differentiation between these species that were previously considered as a single one.

## 3. Experimental Section

### 3.1. Microsatellite-Enriched Library Construction and Isolation of Microsatellite Markers

Genomic DNA was extracted from an individual of *P. contract*a using the Nucleo Spin Plant II kit (Macherey-Nagel, Düren, Germany), and the repetitive motifs were isolated using an enrichment technique [7] in which the genomic DNA was digested with *Rsa*I and *Hae*III and the resulting fragments were linked to two oligonucleotide adaptors. Biotinylated oligonucleotide probes (dGT)_10_, (dGA)_10_, (dAGAT)_10_, (dAACT)_10_, and (dACAT)_10_ were hybridized with the digested DNA and selectively restrained by streptavidin magnetic particles (Promega, Madison, Wisconsin, USA). The selected DNA fragments were eluted in 25 μL ultra pure water and amplified by PCR in a total volume of 50 μL. Reactions were conducted with 50 ng of eluted DNA, 1× Colourless GoTaq Reaction Buffer (Promega), 200 mM dNTPs (Promega), 40 pmol of “oligo adapter A” as primer (Sigma-Aldrich, St. Louis, MO, USA), 1.5 mM MgCl_2_, 5 U of GoTaq Flexi DNA Polymerase (Promega). The PCR conditions were as follows: An initial denaturation at 94 °C for 5 min followed by 35 cycles of denaturation at 94 °C for 1 min, annealing at 56 °C for 1 min and extension at 72 °C for 2 min, with a final extension at 72 °C for 5 min. The enriched library was purified using an UltraClean 15 DNA Purification Kit (MO BIO Laboratories, Carlsbad, CA, USA), linked to the pCR 2.1-TOPO vector (Invitrogen, Carlsbad, CA, USA) and transformed into One Shot TOP10 Chemically Competent Cells (Invitrogen). The plasmid DNA was PCR-amplified using 16 pmol M13(-20) forward and M13(-40) (Sigma-Aldrich), reverse primers, 2.5 U GoTaq Flexi DNA polymerase (Promega), 200 μM of each dNTP (Promega), 2.5 mM MgCl_2_ (Promega), 1× GoTaq Colourless Reaction Buffer (Promega), and 1 μL of transformed cells grown in 100 μL liquid broth LB. The PCR conditions were as follows: An initial denaturation at 94 °C for 5 min, followed by 40 cycles of denaturation at 94 °C for 1 min, annealing at 55 °C for 1 min, and extension at 72 °C for 3 min, with a final extension at 72 °C for 5 min. A total of 163 positive PCR fragments were purified and sequenced using a MegaBACE™ 1000 automated sequencer (GE Healthcare Biosciences, Pittsburgh, PA, USA), following the DYEnamic™ ET terminator sequencing premix kit with terminal fluorescent labeled protocol according to the conditions were as follows: 4 μL of DYEnamic™ ET terminator sequencing premix, 5 μM of forward/reverse primer, 40 ng of purified PCR products, and ultra pure water to complete a 10 μL volume. This reaction was submitted to 95 °C for 20 s, 50 °C for 15 s and 60 °C for 1 min. A total of 23 clones presented perfect unique microsatellites, but only 12 were suitable for primer design using Primer 3 software [8].

### 3.2. Genotyping and Data Analysis

The primers were tested for amplification in two wild populations of *P. contracta* species, Linhares-ES (19°24′S, 40°28′W), *n* = 20 and Maraú-BA (14°07′50″S, 38°59′55″W), *n* = 20, and 10 individuals of *P. ovalis* were tested for cross-amplification. The amplifications were performed in a 15 μL reaction containing ~10 ng template DNA, 1× Taq Platinum reaction buffer (Invitrogen), 200 μM each dNTP (Invitrogen), 2 pmol FAM fluorescently labeled M13(-21) primer [9] and reverse primer, 0.4 pmol forward primer with a 5′-M13(-21) tail, 2.0 mM MgCl_2_ (Invitrogen), and 0.5 U Taq Platinum DNA polymerase (Invitrogen). The PCR conditions for SSR were as follows: An initial denaturation at 94 °C for 3 min, followed by 35 cycles of denaturation at 94 °C for 20 s, annealing at primer-specific temperatures (50–55 °C) see (Table 2) for 45 s, and extension at 72 °C for 1 min, with a final extension at 72 °C for 15 min. The repeat motif, primer sequences, labeling dye, annealing temperature (*T*a °C), and allele size range in base pair, with the M13 tail included, of each primer pair are listed in Table 1.

The fragments were analyzed using MegaBACE™ 1000, based on the ET-ROX 550 size ladder (GE Healthcare). The fragment length and microsatellite genotyping were determined using GENETIC PROFILER 2.0 (GE Healthcare). The allele numbers, expected and observed heterozygosity, Hardy-Weinberg Equilibrium (HWE), and genotypic disequilibrium analyses were performed using ARLEQUIN version 3.5 [10] and FSTAT [11]. MICRO-CHECKER 2.2.3 [12] was used to test for null alleles.

## 4. Conclusions

The development of polymorphic microsatellite markers will contribute to the population genetic studies of *P. contracta*, particularly with regard to comparative studies of diversity patterns in the Atlantic Rainforest. These markers may also help to establish strategies for the conservation of priority population groups of this species that inhabits an extremely endangered ecosystem.

## Figures and Tables

**Table 1:** Characteristics of seven microsatellite markers for *Passiflora contracta*. For each locus, the name, repeat motif, primer sequence, labeling dye, annealing temperature (*T*a), allele size range (bp) and GenBank accession number are shown.

Locus	Repeat motif	Primer sequence (5′–3′)	Dye	*T*a (°C)	Size (bp)	Genbank
PC5E11	(AC)_7_(AG)_6_	F:CTGGTCTTGGATTGTCCTTTG R:CAAAGTAACTGGTGAGCTTAGGG	FAM	54	158–170	JX575753
PC6E8	(GT)_8_	F:TTGCAAATGATAACAAAACACG R:TATCTCGGATTCCCAAAACC	FAM	53	165–181	JX575754
PC6G11	(GA)_10_	F:ACTGGAAGTCAAACGGTGAG R:GGTGGCTCGAAATTCAAATC	FAM	52	207–229	JX575755
PC7H11	(CTT)_13_	F:TGAAATCCCTGTTGTGTGACTC R:TCCTGAGGGGAGCTGTAGTG	FAM	53	169–175	JX575759
PC6D6	(CT)_9_	F:TTTTTGTGAAGGTAATTTGTCA R:CATGTTGCCTCCATGTTTGA	FAM	50	162–168	JX575757
PC7C12	(AC)_7_	F:TGAAATCCCTGTTGTGTGACTC R:TCCTGAGGGGAGCTGTAGTG	FAM	55	179–195	JX575758
PC6F7	(CT)_8_	F:AACGCATTTTTCAGTTTCTGC R:TGAGACTCCCATTCACCAAG	FAM	53	230–248	JX575756

**Table 2:** Characterization of microsatellite loci indicating number of alleles per locus (A); expected (*H*_E_) and observed (*H*_O_) heterozygosity for the two analyzed populations, Linhares-ES (19°24′S, 40°28′W) and Maraú-BA (14°07′50″S, 38°59′55″W), of *Passiflora contracta.*

	*Passiflora contracta*					
	
Locus	Linhares-ES			Maraú-BA		
		
	A	*H*_E_	*H*_O_	A	*H*_E_	*H*_O_
PC5E11#	6	0.82011	0.64286	5	0.63678	0.60000
PC6E8	2	0.31452	0.37500	5	0.46508	0.55556
PC6G11#	4	0.37619	0.44444	8	0.58571	0.44444
PC7H11	3	0.60484	0.75000	3	0.65238	0.38889*
PC6D6	4	0.64368	0.53333	4	0.64308	0.53846
PC7C12	7	0.79637	0.68750	5	0.67137	0.56250
PC6F7	9	0.84135	0.41176*	4	0.57619	0.50000
mean	5			4.8		

# disequilibrium linkage after Bonferroni correction to Linhares-ES population (*p* < 0.002);

* deviation from Hardy-Weinberg equilibrium (*p* < 0.007).
